# In Vitro Effects of Vaspin on Porcine Granulosa Cell Proliferation, Cell Cycle Progression, and Apoptosis by Activation of GRP78 Receptor and Several Kinase Signaling Pathways Including MAP3/1, AKT, and STAT3

**DOI:** 10.3390/ijms20225816

**Published:** 2019-11-19

**Authors:** Patrycja Kurowska, Ewa Mlyczyńska, Monika Dawid, Małgorzata Opydo-Chanek, Joelle Dupont, Agnieszka Rak

**Affiliations:** 1 Department of Physiology and Toxicology of Reproduction, Institute of Zoology and Biomedical Research, Jagiellonian University in Krakow, 30-387 Krakow, Poland; patrycja.kurowska@doctoral.uj.edu.pl (P.K.); ewa.mlyczynska@student.uj.edu.pl (E.M.); monika.dawid@student.uj.edu.pl (M.D.); 2 Department of Experimental Hematology, Institute of Zoology and Biomedical Research, Jagiellonian University in Krakow, 30-387 Krakow, Poland; malgorzata.opydo-chanek@uj.edu.pl; 3 INRA, UMR85, Unité Physiologie de la Reproduction et des Comportements, F37380 Nouzilly, France; joelle.dupont@inra.fr

**Keywords:** vaspin, proliferation, cell cycle, apoptosis, granulosa cells, ovary, pig

## Abstract

Vaspin, a visceral adipose tissue-derived serine protease inhibitor, is expressed in the porcine ovary; it induces the activation of various kinases and steroidogenesis. The aim of this study was to examine the effect of vaspin on granulosa (Gc) proliferation, cell cycle regulation, and apoptosis. Porcine Gc was incubated with vaspin (0.01–10 ng/mL) for 24 to 72 h, proliferation was measured using alamarBlue assay, cell cycle progression was assessed using flow cytometry, and cyclin (D, E, and A) protein expression was measured using immunoblotting. Apoptosis was assessed by measuring caspase activity using Caspase-glo 3/7 assay. Furthermore, histone-associated DNA fragments levels were measured using a cell-death detection ELISA; BAX (bcl-2-like protein 4), BCL2 (B-cell lymphoma 2), caspases (-3, -8, and -9), p53 mRNA, and protein expression were assessed using real time PCR and immunoblotting. We found that vaspin significantly enhanced Gc proliferation and cell cycle progression into the S and G2/M phases and decreased apoptosis. We observed that siRNA silencing of the glucose-regulated protein (GRP78) receptor and pharmacological inhibitors of mitogen-activated kinase (MAP3/1/ERK1/2), Janus kinase (STAT3) and protein kinase B (AKT) blocked the ability of vaspin cell proliferation and enhanced caspase-3/7 activities. These results suggest that vaspin via mitogenic effect on porcine Gc acts as a new regulator of ovarian growth, development, or folliculogenesis.

## 1. Introduction

Mammalian folliculogenesis is involved in follicular growth and follicular atresia and includes processes such as the proliferation, cell cycle control, and differentiation of granulosa cells (Gc). However, only a few follicles undergo ovulation and the majority of follicles are lost before ovulation by atresia. This degenerative process is initiated by Gc apoptosis [1]. It is well known that both proliferation and apoptosis are naturally occurring processes in Gc and are associated with oogenesis, folliculogenesis, oocyte loss/selection, and atresia. An imbalance between these processes can lead to ovarian pathologies. Control of cell proliferation generally occurs during the first gap phase (G1) of the eukaryotic cell division cycle. Multiple signals, ranging from growth factors to DNA damage to developmental cues, influence the decision to enter the S phase, the phase in which DNA is replicated [2]. Moreover, cell cycle peptides, especially cyclins, are involved in the processes of ovarian cell proliferation, growth, and development [3,4,5]. Cell apoptosis is activated by two distinct pathways: extrinsic (or death receptor pathway) and intrinsic (or mitochondrial pathways). Both the intrinsic and extrinsic pathways activate caspases and lead to mitochondrial membrane permeabilization, chromatin condensation, DNA fragmentation, and finally cell destruction [6]. In mitochondrial pathways, some molecules are involved in the process of ovarian follicle atresia (B-cell lymphoma 2 (BCL2) family members and caspases), follicle selection (BCL2, BAX (bcl-2-like protein 4), and caspases), or luteolysis (caspase-3 and BAX) [6]. Knowledge about the molecular mechanism for the regulation of cell proliferation and apoptosis is important for understanding ovarian growth and development as well as folliculogenesis. It is well known that Gc proliferation and apoptotic processes activate multiple signalling pathways including mitogen-activated kinase (MAP3/1/ERK1/2) [7], Janus kinase (STAT3/JAK) [8], protein kinase B (AKT) [9], and 5′ AMP-activated protein kinase (AMPK, know like PRKAA1) [10,11] pathways. For example, AKT provides cells with a survival signal that allows them to withstand apoptotic stimuli [9], while JAK activation stimulates cell proliferation, differentiation, cell migration, and apoptosis [8]. Moreover, substantial evidence has accumulated to indicate an essential role for the MAP3/1 cascade in ovulation, including cumulus cell–oocyte complex (COC) expansion, oocyte maturation, and follicle rupture [12,13]. Reports in the literature have shown that adipokines—hormones produced by adipose tissue—can regulate ovarian cells proliferation and apoptosis in different models, including swine. For example, in vitro studies using porcine ovarian cells showed an inhibitory effect of resistin on cell apoptosis by decreasing caspase activity and the ratio of BAX/BCL2 proteins via the activation of MAP3/1, STAT3, and AKT signalling pathways [14]. 

Vaspin, a visceral adipose tissue-derived serine protease inhibitor, was discovered in 2005 in visceral white adipose tissue of Otsuka Long-Evans Tokushima fatty (OLETF) rats, which are an animal model for the study of obesity and type 2 diabetes [15]. Rat, mouse, and human vaspins are composed of 392, 394, and 395 amino acids, respectively. They are 47-kDa proteins with 40% homology to α1-antitrypsin. Expression of vaspin has been observed mainly in adipose tissue, but it has also found in the liver, pancreas, cerebrospinal fluid, hypothalamus, intestine, and lungs and in human, mouse, bovine, and pig ovaries [16,17,18,19]. Vaspin has pleiotropic functions that include regulating inflammatory responses, insulin resistance and the development of obesity [20]. In endothelial cells, vaspin acts as a ligand for the cell-surface anion channel complex-binding immunoglobulin protein (GRP78)/voltage-dependent [21], which has been shown to be involved in reproduction, cancer progression, and neurologic disorders through the activation of a variety of signalling pathways [22]. Reports have also indicated that vaspin regulates cell proliferation and apoptosis by binding with the GRP78 receptor to induce proliferation and to inhibit apoptosis by the activation of AKT kinase phosphorylation in human endothelial cells [21]. Moreover, vaspin acts as an antiapoptotic agent in cardiomyocytes by attenuating the tumor necrosis factor (TNF-α)-induced apoptosis by promoting autophagy in cardiomyocyte H9C2 cells [23]. Also, vaspin has been shown to inhibit the progression of atherosclerotic plaques in apoE (−/−) mice by inhibiting endoplasmic reticulum stress-induced macrophage apoptosis [24]. Antiapoptotic effects of vaspin have also been described in human osteoblast cells by upregulation of the expression of BCL2 and downregulation of BAX via the MAP3/1 kinase pathway [25] and in mice pulmonary endothelial cells acting by regulating AKT [26]. Vaspin, like other adipokines including leptin, resistin, apelin, or adiponectin, can affect the female reproduction system and locally affects ovarian steroid synthesis and proliferation in women and animal models [27,28]. For example, resistin has an inhibitory effect on the secretion of progesterone (P4) and estradiol (E2) by human luteinized Gc in vitro [29], while adiponectin increased significantly P4 secretion by stimulatory effect on steroidogenic acute regulatory protein (StAR) and cholesterol side-chain cleavage enzyme (CYP11A1) expression in geese ovarian Gc [30]. Our last study showed that, in porcine ovarian follicles, vaspin protein expression was detected in oocyte, Gc, and theca cells and that its level was dependent on fat content and the oestrous cycle [19]. Additionally, several hormones like insulin, insulin-like growth factor type 1 (IGF1), gonadotropins, and steroids increased the ovarian expression of vaspin by the activation of various kinases including MAP3/1, STAT3, AKT, and PRKAA1. In vitro studies using ovarian cells showed that vaspin significantly stimulated steroid synthesis by increasing expression of the steroidogenic enzymes aromatase and 3β-Hydroxysteroid dehydrogenase via activation of the GRP78 receptor and protein kinase A (PKA) pathways [31]. However, the effect of vaspin on ovarian cell proliferation and apoptosis has not been determined.

Thus, the aim of this study was to investigate (i) the dose- and time-dependent in vitro effects of vaspin on Gc proliferation; (ii) the effect of vaspin on cell cycle regulation and cyclin D, E, and A protein expression; (iii) dose- and time-dependent effects of vaspin on apoptosis by evaluating caspase-3 and -7 enzyme activity, levels of histone-associated DNA fragments, and mRNA and protein expression of caspase-3, -8, and -9 and BAX/BCL2 as well as p53; and (iv) the involvement of the GRP78 receptor and kinases MAP3/1, AKT, STAT3, and PRKAA1 on vaspin-mediated Gc proliferation and apoptosis.

## 2. Results

### 2.1. Dose- and Time-Dependent Effects of Vaspin on Gc Proliferation

**Figure 1:**
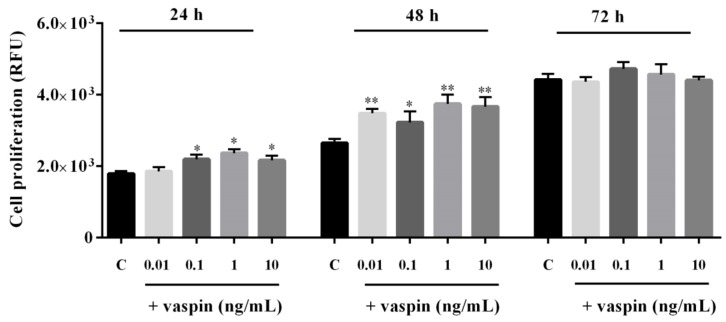
Dose- and time-dependent effect of vaspin on granulosa (Gc) proliferation: Gc were seeded in 96-well culture plates in M199 medium at a concentration of 5 × 10^4^ viable cells per well. The cells were treated with 0.01, 0.1, 1, and 10 ng/mL vaspin for 24, 48, and 72 h. After which, cell proliferation was analysed using the alamarBlue assay. Experiments were independently performed and repeated five times (*n* = 5). The data are plotted as the mean ± SEM. Significance between control and vaspin treatments is indicated by * *p* < 0.05, ** *p* < 0.01; Control (C), Relative Fluorescence Unit (RFU).

Vaspin is known to exert a proliferative effect on several types of cells including human endothelial cells and mouse pulmonary endothelial cells [21,26]. Therefore, we investigated the dose- and time-dependent effects of vaspin on porcine Gc proliferation. As shown in Figure 1, we observed that vaspin significantly stimulated cell proliferation after 24 h of incubation at doses 0.1, 1, and 10 ng/mL. Furthermore, after 48 h of incubation, all doses tested significantly stimulated cell proliferation (* *p* < 0.05, ** *p* < 0.01). However, we documented no differences in Gc proliferation between control and vaspin-treated cells after incubating for 72 h (Figure 1).

### 2.2. Effect of Vaspin on Gc Cycle Regulation

**Figure 2:**
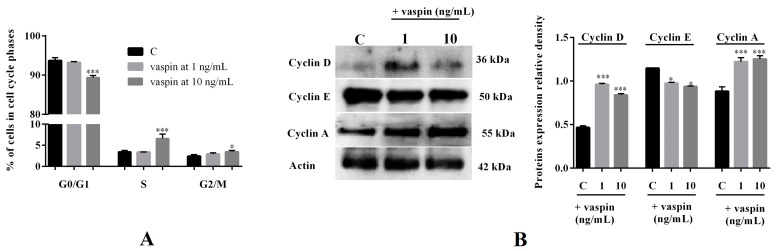
Effect of vaspin on granulosa cell cycle regulation: Cells were treated with vaspin at doses of 1 and 10 ng/mL for 24 h, after which the percentage of Gc in each phase of the cell cycle was measured using flow cytometry (**A**) and the evaluation of cyclins D, E, and A protein expression using Western blot were normalized to the actin levels (full gel images available in the Supplementary Materials) (**B**). Experiments were independently performed and repeated four times (*n* = 4). The data are plotted as the mean ± SEM. Significant differences between control and vaspin treated cells is indicated by * *p* < 0.05, *** *p* < 0.001; Control (C).

To confirm the influence of vaspin on Gc proliferation, we investigated the effect on cell cycle regulation. Our results showed that vaspin at a dose of 10 ng/mL significantly increased the percentage of cells in the S and G2/M phases of cell cycle after 24 h of incubation with a concomitant reduction of cells in the G0/G1 phase of the cell cycle (* *p* < 0.05, *** *p* < 0.001, Figure 2A). As shown in Figure 2B, we observed that 24 h of incubation with both 1 and 10 ng/mL vaspin significantly increased cyclin D and A protein expression and decreased cyclin E protein expression (* *p* < 0.05, *** *p* < 0.001).

### 2.3. Involvement of the GRP78 Receptor and MAP3/1, AKT, STAT3, and PRKAA1 Kinases in Proliferative Effect of Vaspin on Gc Proliferation

**Figure 3:**
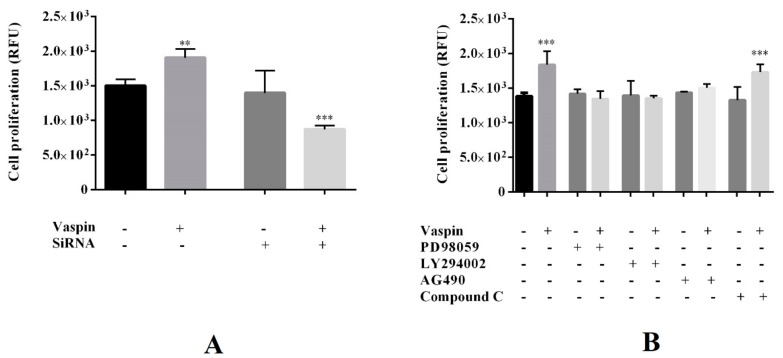
Involvement of glucose-regulated protein (GRP78) receptor (**A**) and mitogen-activated kinase (MAP3/1/ERK1/2), Janus kinase (STAT3) and protein kinase B (AKT) and 5′ AMP-activated protein kinase (AMPK, know like PRKAA1) kinases (**B**) on vaspin-mediated induction of granulosa (Gc) proliferation: Gc were seeded in 96-well culture plates in M199 medium at a concentration of 5 × 10^4^ viable cells per well. Cells were pretreated with GRP78 siRNA (2 nM) for 24 h or for 1 h with the following MAP3/1, AKT, STAT3, and PRKAA1 kinases inhibitors: PD098059 (5 µM), LY294002 (10 μM), AG490 (50 μM), and Compound C (1μM). Afterward, vaspin (1 ng/mL) was added for 24 h and cell proliferation was analysed using an alamarBlue assay. Experiments were independently performed and repeated five times (*n* = 5). The data are plotted as mean ± SEM. Significance between control and vaspin or inhibitors and vaspin + inhibitors treatments are indicated by ** *p* < 0.01 and *** *p* < 0.001; Control (C), Relative Fluorescence Unit (RFU).

Previously, data has shown that vaspin promotes the expression of the GRP78 receptor and the phosphorylation of MAP3/1, AKT, STAT3, and PRKAA1 kinases in porcine ovarian follicles [31]. Therefore, in the present study, we investigated the role of the GRP78 receptor and investigated MAP3/1, AKT, STAT3, and PRKAA1 kinases on vaspin-mediated induction of Gc proliferation. Cell proliferation was assessed after 24 h incubation with GRP78 siRNA (2 nM) or pharmacological inhibitors PD098059 (5 µM), LY294002 (10 μM), AG490 (50 μM), and Compound C (1μM) of the MAP3/1, AKT, STAT3, and PRKAA1 kinases, respectively. As shown in Figure 3, we observed that simultaneous treatment with PD098059, LY294002, or AG490 added with vaspin (1 ng/ml) reversed cell proliferation to control levels, while simultaneous treatment of GRP78 and vaspin significantly reduced Gc proliferation compared to control (untreated cells) or siRNA (** *p* < 0.01, *** *p* < 0.001). Additionally, we noted that, in Gc cultures treated with both Compound C and vaspin, the levels of proliferation of cells was significantly higher, similar to that in culture with vaspin alone (*** *p* < 0.001). We also observed that, when added alone, GRP78 siRNA and each of the inhibitors used did not affect cell proliferation (Figure 3).

### 2.4. Effect of Vaspin on Caspase-3 and -7 Activity and Histone-Associated DNA Fragment Levels in Gc

**Figure 4:**
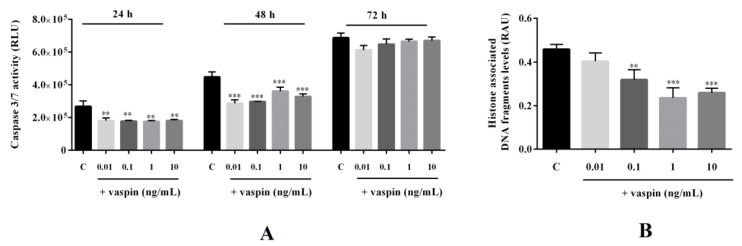
Effect of vaspin on caspase-3 and -7 activity and histone-associated DNA fragment levels: The cells were treated with 0.01, 0.1, 1, and 10 ng/mL vaspin for 24, 48, and 72 h, after which caspase-3 and -7 activity (**A**) was analysed using a Caspase-glo 3/7 assay or were incubated for 24 h to study levels of histone-associated DNA fragments (**B**) using a cell-death detection kit. Experiments were independently performed and repeated five times (*n* = 5). The data are plotted as means ± SEM. Significant differences between control and vaspin treatments are indicated by ** *p* < 0.01 and *** *p* < 0.001; Control (C), Relative Luminescence Unit (RLU), Relative Absorbance Unit (RAU).

Vaspin also has antiapoptotic properties in different cell types including human osteoblasts and rat pancreatic cells [25,32]. Therefore, we investigated the dose- and time-dependent effects of vaspin on porcine Gc apoptosis. As shown in Figure 4, we observed that vaspin significantly decreased caspase-3 and -7 activity after 24 h and 48 h of incubation in all studied doses 0.01, 0.1, 1, and 10 ng/ml, while it has no effect after 72 h of incubation (** *p* < 0.01, *** *p* < 0.001, Figure 4A). Next, we observed that vaspin at doses 0.1, 1, and 10 ng/mL significantly decreased levels of histone-associated DNA fragments (** *p* < 0.01, *** *p* < 0.001, Figure 4B).

### 2.5. Effect of Vaspin on mRNA and Protein Expression of Caspases-3,-8, and -9; BAX; BCL2; and p53 in Gc

**Figure 5:**
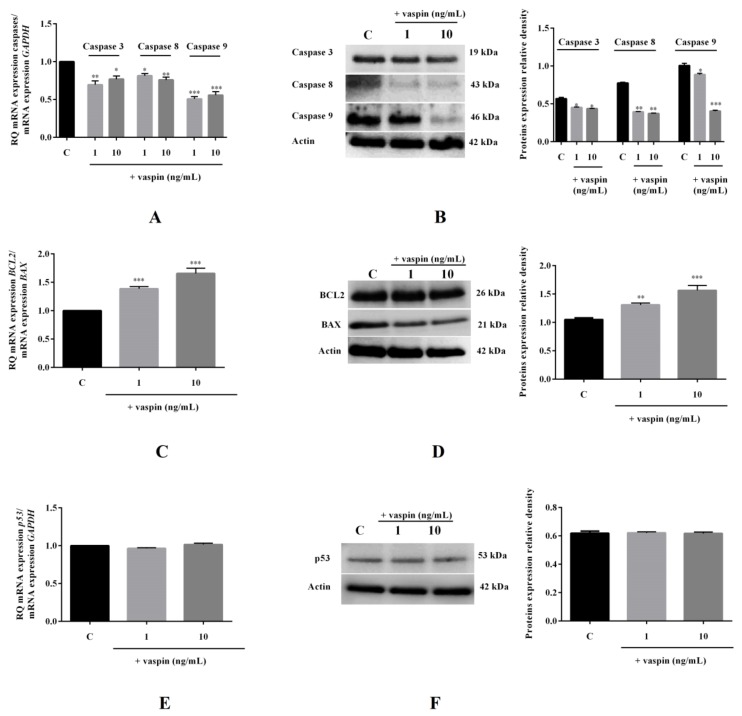
Effect of vaspin on mRNA and protein expression levels of caspase-3, -8, and -9; BAX (bcl-2-like protein 4), and BCL2 (B-cell lymphoma 2). The cells were treated with 1 and 10 ng/mL vaspin for 24 h, and then, real-time PCR and Western blot analysis were performed to determine levels of caspase-3, -8, and -9 (**A**,**B**); BCL2 and BAX (**C**,**D**); and p53 (**E**,**F**) (full gel images are available in the Supplementary Materials). Gene expression levels were normalised to glyceraldehyde 3-phosphate dehydrogenase (*GAPDH)*, while proteins levels were normalized to actin. Experiments were independently performed and repeated five times (*n* = 5). The data are plotted as the means ± SEM. Significance between control and vaspin treatments is indicated by * *p* < 0.05, ** *p* < 0.01 and *** *p* < 0.001; Control (C).

We found that vaspin at doses 1 and 10 ng/mL significantly decreased caspase-3 (1.4- and 1.3-fold), caspase-8 (1.2- and 1.3-fold), and caspase-9 (2.0- and 1.8-fold) mRNA expression fold to control (1). Similar effects were observed regarding the protein expression of caspases (* *p* < 0.05, ** *p* < 0.01, *** *p* < 0.001, Figure 5A,B). Furthermore, we observed that vaspin at 1 and 10 ng/mL significantly increased the BCL2/BAX ratio of mRNA expression by 1.4- and 1.7-fold compared to the control ratio (1), respectively, as well as the ratio of BCL2/BAX protein expression (** *p* < 0.01, *** *p* < 0.001, Figure 5C,D). We demonstrated that vaspin has no effect on p53 expression at both the mRNA and protein levels (Figure 5E,F).

### 2.6. Involvement of the GRP78 Receptor and MAP3/1, AKT, STAT3, and PRKAA1 Kinases in the Antiapoptotic Effect of Vaspin in Gc

**Figure 6:**
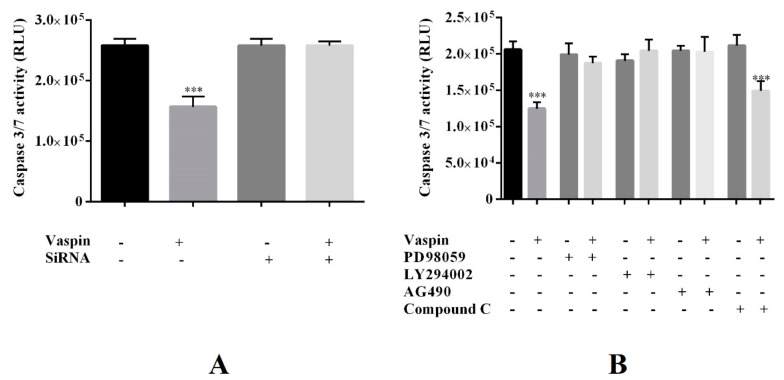
Involvement of the GRP78 receptor (**A**) and MAP3/1, AKT, STAT3, and PRKAA1 kinases (**B**) in vaspin-mediated Gc apoptosis: Cells were pretreated with GRP78 siRNA (2 nM) for 24 h or were treated for 1 h with the following MAP3/1, AKT, STAT3, and PRKAA1 kinase inhibitors: PD098059 (5 µM), LY294002 (10 μM), AG490 (50 μM), and Compound C (1μM). Vaspin (1 ng/mL) was added for 24 h. Caspase-3 and -7 activities were analyzed using the Caspase-Glo 3/7 assay. Experiments were independently performed and repeated five times (*n* = 5). Data are plotted as the means ± SEM. Significant differences between control and vaspin treatments or inhibitor and vaspin + inhibitors treatment are indicated by *** *p* < 0.001; Control (C), Relative Luminescence Unit (RLU).

As shown in Figure 6, we observed that both GRP78 siRNA and inhibitors PD098059, LY294002, and AG490, when added simultaneously with vaspin (1 ng/mL), reversed caspase-3 and -7 activity to the control levels. However, we observed that caspase-3 and -7 activity was decreased in Gc treated with the PRKAA1 kinase inhibitors—Compound C and vaspin. Levels were similar to those produced by treatment with vaspin alone (*** *p* < 0.001, Figure 6). In addition, we observed that GRP78 siRNA and all investigated inhibitors added alone had no effect on caspase activity.

## 3. Discussion

In the present study, we have demonstrated that vaspin increased Gc proliferation and cell cycle progression into the S and G2/M phases by stimulation of the protein expression levels of cyclins A and D. Our results also have shown that vaspin significantly decreased cell apoptosis by directly affecting the enzymatic activities of caspase, DNA fragmentation, and mRNA and protein expression of caspase and BAX/BCL2. No effect of vaspin on p53 level was observed. Finally, we documented that the GRP78 receptor kinases MAP3/1, AKT, and STAT3 are involved in both the proliferative and antiapoptotic effects of vaspin in porcine Gc.

Proliferation is an important process in ovarian physiology and participates in folliculogenesis and oocyte growth [6]. During follicle formation, Gc change their morphological and physiological properties, changes that are connected to cell proliferation. Abnormalities in folliculogenesis can led to ovarian pathologies like infertility, polycystic ovarian syndrome, and the generation of cancer [33]. The results of our in vitro studies clearly show that vaspin, at all investigated doses ranging from 0.01 to 10 ng/mL, stimulated Gc proliferation after incubation for 24 and 48, but not 72 h, suggesting that there is a time-dependent period in which vaspin stimulates proliferation. The experiments reported here used physiological doses of vaspin, which have been shown to be 1 ng/mL in women serum [34] or porcine plasma and follicular fluid [19]. Our results are in agreement with previous reports in the literature that have shown that 10 ng/mL vaspin stimulated proliferation in human aortic endothelial cells by binding the GRP78 receptor and by enhancing activation of the AKT kinase pathway [21]. The results of our study suggest that vaspin, like other hormones including E2, folliculotropin (FSH), oxytocin, and IGF1 can promote porcine ovarian follicle growth by enhancing cell proliferation [35]. The proliferative effect of vaspin in Gc occurred in parallel with the promotion cell cycle progression into the S and G2/M phases. Progression throughout the G2/M phase is accomplished by the expression of cyclin A, in which we observed stimulatory effect of vaspin on protein expression. Cyclin A begins to appear toward the end of G1, and levels of the protein continue to rise throughout the S phase. Then, levels rapidly decline as the cell progresses through G2 [36]. Our results indicate that vaspin can promote DNA synthesis and can trigger a transition from S phase to G2/M phase. Moreover, we have been able to show increased levels of cyclin D protein expression, while cyclin E levels deceased, associated with an increase in the percentage of Gc in the S and G2/M phases of cell cycle. The observed effect indicates that vaspin facilitates Gc cell transition from the G0/G1 phase to the S and G2/M phases of cell cycle. Importantly, this effect was seen when vaspin was used at a higher dose of 10 ng/mL which indicates that vaspin influences cell cycle progression dose-dependently. Enhanced levels of cyclin D expression is followed during G1 by the appearance of cyclin E, which quickly decreases at the beginning of DNA synthesis (S phase) [36]. The canonical Ras–Raf–MEK–ERK–MAPK pathway is the best characterized pathway for the activation of cyclin D transcription [37]. It stimulates the expression of Activator protein 1 (AP1) transcription factors such as the proto-oncogene products Jun and Fos, which bind directly to an AP1 site in the cyclin D1 promoter; however, in our experiments, we cannot study the involvement of kinases in vaspin effect on cyclins expression. It is well known that cyclins are regulators of key events during the progression of the cell cycle [38] as well as are established markers of porcine ovarian cell proliferation, growth, and development [39,40]. Cell cycle regulators are important for the regulation of proliferation, and the results of our studies clearly suggest that the promotion of cell proliferation by caspases is mediated by vaspin. Our results are in agreement with Han et al. [41], who indicated that FSH increased cyclin D protein expression, which is connected with the stimulation of proliferation via activation of PKA and AKT kinases in rat Gc. Moreover, other adipokines, such as leptin, chemerin, resistin, and apelin, can affect Gc proliferation in different species [14,42,43,44,45,46,47,48].

To investigate the molecular mechanism involved in the mitogenic action of vaspin in Gc, we transfected cells with GRP78 siRNA and inhibited by using kinases MAP3/1, AKT, STAT3, and PRKAA1, which are pharmacological inhibitors. These signalling pathways have been shown to play a role in Gc proliferation in response to various hormones such as FSH, IGF1, and insulin, as well as, more recently some adipokines including leptin, apelin, and adiponectin [47,49,50,51,52,53,54]. Interestingly, we noted that cells transfected with GRP78 siRNA or with MAP3/1, AKT, and STAT3 inhibitors effectively blocked vaspin-induced cell proliferation while did not affect the proliferation of cells treatment with PRKAA1 kinase inhibitor. Gc proliferation is controlled by a complex signalling network [55]. This analysis showed that MAP3/1, AKT, and STAT3 signalling pathways were correlated with Gc proliferation 24 h following stimulation with vaspin. MAP3/1, AKT, or STAT3 pathways have been reported to affect Gc proliferation by regulating the cell cycle and are associated with the actions of growth factors or other adipokines. Previous studies have shown that MAPK/ERK and Akt/PI3 kinases mediate the proliferative effects of apelin in porcine ovarian cells [47] and rat Gc [56]. Our previous work showed that vaspin significantly increased levels of PRKAA1 kinase phosphorylation in ovarian cells [31]; however, in the present study, we observed that Gc proliferation induced by vaspin did not involve this kinase, suggesting that vaspin may regulate other processes in ovarian cells via activation of PRKAA1 kinase. It is well known that AMPK (a known PRKAA1 kinase) plays an important role not only in the proliferation and survival of somatic gonadal cells but also in modulation of gonadal steroidogenesis and oocytes maturation [57]. Whether PRKAA1 kinase participates in vaspin-induced ovarian cell regulation remains to be determined.

The results of our study have shown that stimulation of Gc proliferation may be due to decreased cell apoptosis, an important process in the ovary linked with oocyte growth, selection to ovulation, and atresia [6]. We observed that vaspin significantly decreased caspase-3, -8, and -9 expression and enzyme activities of caspase-3/-7, suggesting vaspin action on both extrinsic and mitochondrial pathways of apoptosis. In addition, caspases are key mediators of DNA fragmentation. In our studies, we demonstrated that vaspin significantly decreased levels of histone-associated DNA fragments, confirming the inhibitory effect of vaspin on Gc apoptosis. Moreover, we observed that vaspin altered the ratio of antiapoptotic BCL2 to proapoptotic BAX proteins at both the mRNA and protein levels. In the mitochondria pathway, BAX promotes proapoptotic signals, allowing for the release cytochrome c from mitochondria, while BCL2 inhibits cytochrome c release. Moreover, BCL2 and BAX proteins have a possible role in atresia, since they are expressed in Gc of both foetal and adult ovaries [58]. Expression profiles of the two proteins are different: BAX has been found in atretic follicles, while BCL2 has been observed in growing follicles [59]. These results are in accordance with those reported by Zhu et al. [25], who observed that vaspin increased the ratio of BCL2/BAX in human osteoblasts by activation of the MAP3/1 kinase pathway. On the other hand, our results demonstrated that vaspin has no effect on p53 expression, which is important in ovarian apoptosis—overexpression of p53 can induce apoptosis in cAMP-stimulated cells [6]. Expression of the p53 protein in apoptotic Gc cells of atretic follicles suggests its possible role in atresia [60]. Inhibition of p53 expression is associated with a marked reduction in the number of apoptotic Gc and atretic follicles [61]; however, in our study, no effect of vaspin on p53 level was observed, as in the experiments with resistin in ovarian cells [14]. There is evidence in the literature showing that vaspin plays a key role in the regulation of apoptosis in other cell types including endothelial cells [62], cardiomyocytes [23], and human osteoblasts [25]. Also, vaspin, like other adipokines including leptin, adiponectin, and resistin, regulates ovarian cell apoptosis [14,43,63,64].

Our results are in good agreement with previous studies, which confirmed that ovarian cell survival and apoptosis depended on the activation of various kinases, for example, MAP3/1, AKT, and STAT3 signalling pathways were activated by resistin in porcine ovarian follicles [14] while the AKT signalling pathway participated in luteinizing hormone (LH)-mediated apoptosis suppression in goat theca cells [65]. However, in our study, we observed that vaspin cannot induce PRKAA1 kinase phosphorylation in apoptosis. As we know, signalling pathways participate in cross-talks, which should be considered here. For instance, Akt negatively regulates the function and expression of several proteins, including BAD (BCL2 antagonist of cell death), which has been shown to downregulate proliferation [66]. Akt-mediated phosphorylation of human procaspase-9 and its phosphorylation correlates with decreases in caspase-9 levels. The ERK pathway plays a critical role in promoting several forms of cell death including (i) activation of death receptors by increasing the level of death ligands such as tumor necrosis factor alpha (TNFα) or Fas Ligand (FasL) or death receptors such as Fas, death receptor 4 (DR4) or death receptor 5 (DR5); (ii) activation of initiator caspase-9 or caspase -8; (iii) mitochondria membrane disruption and cytochrome c release; or (iv) upregulation of proapoptotic members of the Bcl-2 family, such as Bax [67]. Interestingly, our results indicate that the GRP78 receptor is involved in the stimulation of cell division in Gc because GRP78 siRNA blocked both vaspin-induced cellular proliferation and the promotion of caspase-3/-7 activity. Previously published data has shown that vaspin binds GRP78 in mice and that the association of vaspin with GRP78 in obesity was associated with metabolic dysfunction that transduces the intracellular signalling such as AKT and AMPK, which results in the improvement of both glucose and lipid metabolism [68]. GRP78 can also induce the activation of other signal transduction pathways, like MAP3/1 and STAT3 [69]. Moreover, GRP78 is associated with various anchor membrane proteins on the cell surface and functions as a receptor for various ligands, functioning as a signalling hub for cell survival and cell death [70,71]. In our study, we investigated the involvement of kinases MAP/1, AKT, STAT3, and PRKAA1 in the vaspin effect on Gc proliferation and apoptosis. However, many other signalling pathways such as cAMP/PKA or NFκB (nuclear factor kappa-light-chain-enhancer of activated B cells) could also contribute to explaining the molecular effect of vaspin in the ovarian cells.

## 4. Materials and Methods

### 4.1. Reagents

Foetal bovine serum (FBS), TaqMan Gene Expression Cells-to-CT Kit (product no. AM1728), electrophoresis marker, and lipofectamine 3000 (product no. L3000001) were purchased from ThermoFisher Scientific (Waltham, MA, USA). Phosphate buffered saline (PBS) was purchased from BioWest (Riverside, MO, USA). Medium M199, antibiotic-antimycotic solution, Tris, human vaspin (product no. SRP4915), Laemmli buffer (product no. 38733), AG490 (product no. T3434), and Compound C (product no. P5499) were obtained from Sigma-Aldrich (St. Louis, MO, USA). Pig vaspin was not available; therefore, human vaspin was used (human and pig vaspin share 90% sequence homology, as determined using the NCBI (USA) database using BLAST program, 2019). PD98059 (product no. 1213) was obtained from Tocris (Bristol, GB); 4–20% gels (product no. 456-1093) and membranes (product no. 1704156) were obtained from BioRad (Hercules, CA, USA). LY294002 (product no. 9901) was obtained from Cell Signalling Technology (Danver, MA, USA).

### 4.2. Gc In Vitro Cultures

Ovaries were collected from mature (6–8 months) Polish large white pigs at days 10–12 of oestrous cycle at a slaughterhouse under veterinarian control. Ovaries were transported to the laboratory in PBS with antibiotic-antimycotic solution. Within 30 min of collection, ovarian follicles were isolated from ovaries after morphological examination [72], and then, Gc were isolated. Gc cultures was prepared according to the technique described by Stoklosowa et al. [73]. Briefly, Gc were scrubbed from the follicular wall with round-tipped, ophthalmologic tweezers and rinsed several times with PBS. After isolation, Gc were exposed to DNAse I (500 U for 1 min), washed three times in M199, collected, and resuspended in M199 supplemented with 10% FBS. The viability of the cells (92%) were determined using the Trypan blue exclusion test. Gc were seeded in 96-well culture plates in M199 medium with 10% FBS for 24 h at a concentration of 5 × 10^4^ viable cells per well. Next, medium was changed to M199 with 1% FBS. All cultures were maintained at 37 °C in a humidified atmosphere consisting of 5% CO_2_/95% O_2_.

#### 4.2.1. Dose- and Time-Dependent Effects of Vaspin on Cell Proliferation and Caspase-3/7 Activity

Gc were incubated for 24, 48, or 78 h in M199 medium supplemented with 1% FBS and vaspin at 0.01, 0.1, 1, and 10 ng/mL. Doses of vaspin were chosen based on previous publications [25,74], where authors studied the effects of various doses of vaspin (0.1, 1, 10, and 100 ng/ml) on apoptosis and differentiation in in vitro human osteoblasts cells. In our previous published data [19], we determined levels of vaspin in plasma and follicular fluid of ovarian follicles collected from different days of the estrous cycle in pigs. We demonstrated that the physiological level of vaspin both in plasma and follicular fluid were in the range of 0.5–1 ng/mL. In another paper [31], we clearly documented that vaspin at doses 0.01, 1, and 10 ng/mL significantly increased steroid secretion and activation of various kinases signalling pathways in porcine ovarian follicular cells. After incubation, alamarBlue reagent was added for 3 h to assess cell proliferation or Caspase-Glo 3/7 reagent was added for 1.5 h to evaluate caspase-3/-7 activity.

#### 4.2.2. Effect of Vaspin on Levels of Histone-Associated DNA Fragments and the Regulation of the Cell Cycle

Gc were incubated for 24 h in M199 medium supplemented with 1% FBS and vaspin at concentrations of 0.01, 0.1, 1, and 10 ng/mL. After incubation, cells were washed in PBS and frozen at −80 °C to evaluate levels of histone-associated DNA fragments using cell-death detection ELISA kit. For cell cycle experiments, Gc were incubated for 24 h in M199 medium with 1% FBS and vaspin at 1 or 10 ng/mL and then cells were fixed with 70% cold ethanol at 4 °C for 60 min and stored at −20 °C for flow cytometry.

#### 4.2.3. Effect of Vaspin on Cyclin Protein Expression as Well Caspases and BAX/BCL2 mRNA and Protein Expression

Gc were incubated for 24 h in M199 supplemented with 1% FBS and vaspin at 1 or 10 ng/mL. After incubation, cells were washed in PBS and boiled in Laemmli buffer for 4 min and stored at −20 °C to evaluate cyclin D, E, and A; caspase-3, -8, and -9; and BAX/BCL2 protein expression level. To analyze mRNA expression, Gc were washed in PBS and frozen in −80 °C for real time PCR.

#### 4.2.4. Assessing the Involvement of the GRP78 Receptor in Vaspin-Mediated Gc Proliferation and Caspase-3/7 Activity

Gc were incubated with M199 medium without FBS for 24 h and then treated with 2 nM GRP78 siRNA. Next, after 24 h of starvation, vaspin was added at a dose 1 ng/mL and cells were incubated for 24 h. Cell proliferation was assessed using alamarBlue assay and caspase-3/7 activities were assessed using a Caspase-Glo 3/7 assay. The concentrations of GRP78 siRNA were chosen based on previous work [31]. 

#### 4.2.5. Involvement of MAP3/1, AKT, STAT3, and PRKAA1 Kinases in Vaspin Induction of Proliferation and Caspase-3/7 Activity

Gc were incubated for 24 h in M199 supplemented with 1% FBS, and cells were pretreated for 1 h with the MAP3/1, AKT, STAT3, and PRKAA1 kinase inhibitors: PD098059 at 5 µM, LY294002 at 10 μM, AG490 at 50 μM, and Compound C at 1 μM, respectively. Then, vaspin (1 ng/mL) was added. The concentrations of the pharmacological inhibitors were chosen based on our previous data [19]. After 24 of Gc incubation, proliferation was measured using alamarBlue reagents and caspase-3/7 activities were assessed using Caspase-Glo 3/7.

### 4.3. Gene Silencing

GRP78 silencing in porcine Gc was designed according to rules described by Park et al. [75]. Gc was transfected with GRP78 siRNA (2 nM) using Lipofectamine 3000 according to the manufacturer’s instructions and previous experiments [31]. The sequences of siRNA against GRP78 used here were #909: CCU UCU CAC CAU UGA UAA UTT (sense), AUU AUC AAU GGU GAG AAG GTT (antisense); #693: GGG AAA GAA GGU UAC UCA UTT (sense), AUG AGU AAC CUU CUU UCC CTT (antisense); and #1570: GCC UCU GAU AAU CAG CCA ATT (sense), UUG GCU GAU UAU CAG AGG CTT (antisense). 

### 4.4. AlamarBlue Assay

The alamarBlue assay (product no. DAL1100, Invitrogen, Carlsbad, CA, USA) assessed minimal toxicity, which was based on the quantitation of cell metabolic activity and allows for the study of cell proliferation. The reaction is based on the metabolic conversion of the blue, nonfluorescent resazurin to a pink, fluorescent resorufin by living cells. AlamarBlue stock solution was aseptically added to wells in amounts equal to 10% of the incubation volume. The resazurin reduction was determined after 3 h incubation with alamarBlue by measuring absorbance at 570 and 600 nm wavelengths using a FLUORO reader (BioTek Instruments, Winooski, VT, USA).

### 4.5. Flow Cytometry

Gc was washed twice in 1 mL PBS. Next, the cell pellet was resuspended in 300 μL of the propidium iodide/RNase staining buffer (BD Biosciences, San Jose, CA, USA) and incubated in the dark for 30 min at room temperature. The red fluorescence of propidium iodide was measured by FACS Calibur flow cytometer (Becton Dickinson, Franklin Lakes, NJ, USA). Ten thousand cells were examined per sample. The percentage of the cell population in each cell cycle phase (G0/G1, S, and G2/M) was calculated from DNA content histograms using the WinMDI 2.8 software.

### 4.6. Caspase-Glo 3/7 Assay

The Caspase-Glo 3/7 assay (product no. G8090, Promega, Madison, WI, USA) results in cell lysis, followed by caspase cleavage of the substrate. This liberates free aminoluciferin, which is consumed by luciferase to generate a “glowing” luminescent signal that is proportional to caspase-3 and -7 activity. The Caspase-Glo 3/7 assay stock solution was aseptically added to wells in amounts equal to 100% of the incubation volume. The luminescence after 1.5 h of incubation was measured at 495 nm wavelength using a luminometre SpectraMax L 147 and SoftMax Pro software (software version 7, Molecular Devices, San Jose, CA, USA).

### 4.7. Cell-Death Detection ELISA Kit

The cell-death detection ELISA kit (product no. 11 544 675 001, Roche Diagnostics, Basel, Switzerland) is an enzyme immunoassay used for the determination of levels of cytoplasmic histone-associated DNA fragments. The assay is based on the quantitative sandwich enzyme immunoassay principle using mouse monoclonal antibodies directed against DNA and histones. The assay was performed according to the manufacture’s protocol. Absorbance was measured at the 405 nm wavelength using an ELx808 ELISA microplate reader and KC JUNIOR software (BioTek Instruments, Winooski, VT, USA).

### 4.8. Real-Time PCR

RNA isolation and cDNA synthesis were performed following the manufacturer’s protocol using TaqMan™ Gene Expression Cells-to-CT™ Kit. RNA and cDNA quantity were evaluated by measuring absorbances at 260 nm and 280 nm using spectrophotometry. Amplifications were performed using the StepOnePlus system (Applied Biosystems, Carlsbad, CA, USA) following the manufacturer’s instructions. TaqMan specific primers and TaqMan Gene Expression Master Mix were used. PCR was performed using a final volume of 20 μL, including 50 ng/reaction cDNA. The relative genes (ThermoFisher Scientific, Waltham, MA, USA) were -caspase 3 (product no. Ss03382792), -caspase 8 (product no. Ss03379427), and -caspase 9 (made to order in Applied Biosystems, based on cDNA sequence described by Matsui et al. [76]; forward: 5′-GGCTG TCTAC GGCAC AGATG GA-3′, reverse: 5′-CTGGC TCGGG GTTAC TGCCA G-3′); and *BAX* (product no. Ss03375842), *BCL2* (product no. Ss03375167), and *p53* (product no. Ss04248637). Expression levels were normalized against reference gene *GAPDH* (product no. Ss03375629).

Quantitative PCR was performed with 100 ng cDNA, 1 mL TaqMan GeneExpression primers, and 10 mL TaqMan PCR master mix (Applied Biosystems) in a final reaction volume of 20 mL. After a 2-min incubation at 50 °C, thermal cycling conditions were 10 min at 95 °C, followed by 40 cycles of 15 s at 95 °C and 1 min at 60 °C to determine the cycle threshold number (Ct) for quantitative measurement. The relative mRNA expression levels of apoptosis genes relative to GAPDH were determined using the 2^−ΔΔCq^ method [77]. 

### 4.9. Western Blot

Western blot and quantification of protein expression were performed as previously described [78]. Proteins were reconstituted directly in appropriate amounts of sample buffer, separated in Mini-Protean TGX System Precast Protein Gels, and then transferred to Trans-Blot Turbo Mini PVDF Transfer membranes. The membranes were washed and blocked in 0.02 M Tris-buffered saline containing 5% bovine serum albumin (BSA) and 0.1% Tween 20 and then incubated overnight at 4 °C with appropriate antibodies (Table 1). Next, the membranes were washed with TBST (Tris-buffered saline containing 0.1% Tween 20) and incubated for 1 h with horseradish peroxidase-conjugated secondary (Table 1). An anti-actin antibody was used as loading control. Signals were detected by chemiluminescence using a WesternBright Quantum horseradish peroxidase (HRP) substrate (product no. K-12043 D20, Advansta Inc., Menlo Park, CA, USA) and visualized using the Chemidoc XRS + System (BioRad, Hercules, CA, USA). All visible bands were quantified using a densitometer and ImageJ software (US National Institutes of Health, Bethesda, MD, USA). 

### 4.10. Statistical Analysis

Statistical data have been presented as means ± standard error of the mean (SEM) of five independent experiments. Distribution of normality was assessed by using the Shapiro–Wilk test. Statistical analysis was carried out using one-way or two-way ANOVA, followed by Tukey’s test (PRISM software version 5; GraphPad, La Jolla, CA, USA). Statistical significance is indicated by * *p* < 0.05, ** *p* < 0.01, and *** *p* < 0.001.

## 5. Conclusions

**Figure 7:**
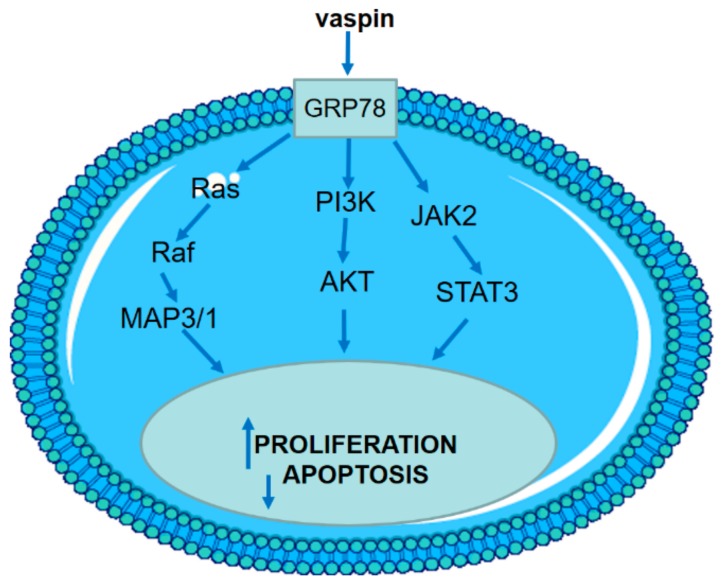
Model of signal-transducing pathways regulating porcine Gc proliferation and apoptosis: Vaspin stimulate proliferation and inhibit apoptosis by binding the GRP78 receptor and by leading to MAP3/1, AKT, and STAT3 kinases activation.

In this study, we evaluated the effect of vaspin on Gc proliferation, cell cycle regulation, and apoptosis. We noted that the stimulatory effect of vaspin on cell proliferation and its inhibitory effect on apoptosis are dependent on the GRP78 receptor and the MAP3/1, AKT, and STAT3 signalling pathways (Figure 7). Vaspin through regulation of cell proliferation and apoptosis control ovarian follicle growth, selection, or atresia as well as folliculogenesis. 

## Figures and Tables

**Table 1:** Antibodies used in Western blot reaction.

Antibody	Host Species	Dilution	Vendor/Product No.
cyclin D	rabbit	1:1000	Cell Signalling Technology, USA, product no. 2978S
cyclin E	mouse	1:1000	Abcam, Cambridge, GB, product no. ab3927
cyclin A	mouse	1:1000	Cell Signalling Technology, USA, product no. 4656S
caspase-3	rabbit	1:1000	Cell Signalling Technology, USA, product no. 9662S
caspase-8	mouse	1:500	ThermoFisher Scientific, USA, product no. MA1-41280
caspase-9	rabbit	1:1000	Invitrogen, Invitrogen, USA, product no. PA5-22252
BCL2	rabbit	1:1000	Cell Signalling Technology, USA, product no. 4223S
BAX	rabbit	1:1000	Cell Signalling Technology, USA, product no. 2772
p53	rabbit	1:1000	Cell Signalling Technology, USA, product no. 9282S
actin	mouse	1:5000	Sigma-Aldrich, USA, product no. A5316
anti-rabbit	goat	1:1000	Cell Signalling Technology, USA, product no. 7074
anti-mouse	horse	1:1000	Cell Signalling Technology, USA, product no. 7076

## Supplementary Materials

Supplementary materials can be found at https://www.mdpi.com/1422-0067/20/22/5816/s1.

## Abbreviations

Gc	Granulosa cells
BCL2	B-cell lymphoma
MAP3/1/ERK1/2	Mitogen-activated kinase
STAT3	Janus kinase
AKT	Protein kinase B
AMPK/PRKAA1	5′ AMP-activated protein kinase
COC	Cumulus cell–oocyte complex
OLETF	Otsuka Long-Evans Tokushima fatty
P4	Progesterone
GRP78	Binding immunoglobulin protein
TNF-α	Tumor necrosis factor
IGF1	Insulin like growth factor type 1
RFU	Relative Fluorescence Unit
RLU	Relative Luminescence Unit
RAU	Relative Absorbance Unit
FSH	Folliculotropin
PCNA	Proliferation-related peptide
LH	Luteinizing hormone
FBS	Foetal bovine serum
PBS	Phosphate buffered saline
Ct	Cycle threshold number
HRP	Horseradish peroxidase
BSA	Bovine serum albumin
E2	Estradiol
