# Supplementary figures and images for: In Vitro Effects of Vaspin on Porcine Granulosa Cell Proliferation, Cell Cycle Progression, and Apoptosis by Activation of GRP78 Receptor and Several Kinase Signaling Pathways Including MAP3/1, AKT, and STAT3
Source: Int J Mol Sci. 2019 Nov 19;20(22):5816. doi: 10.3390/ijms20225816 (PMC6888539; doi:10.3390/ijms20225816)

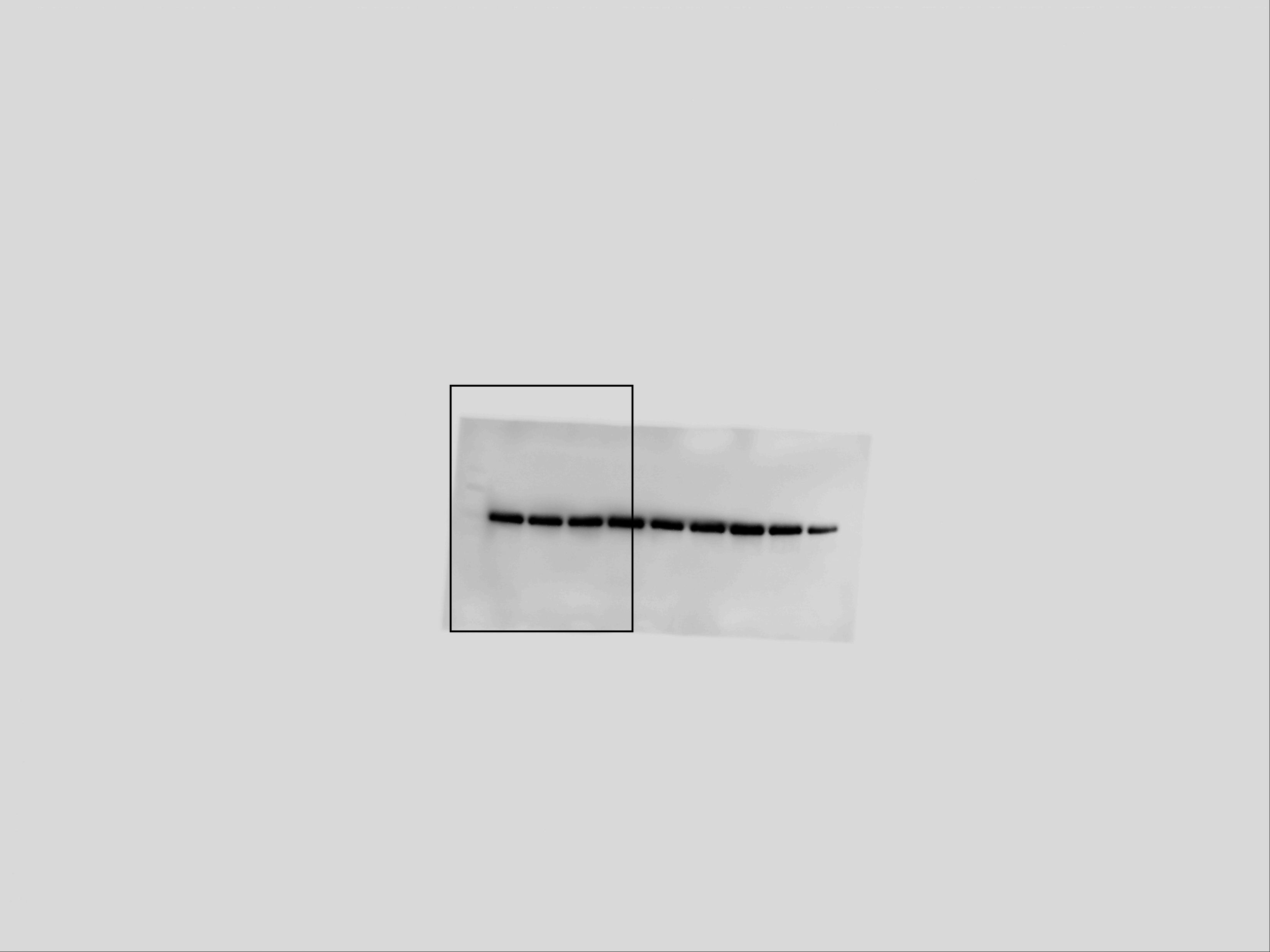

Supplement: Supplementary file 1 [file ijms-20-05816-s001.zip › ijms-618561-supplementary/Actin for p53.JPG]

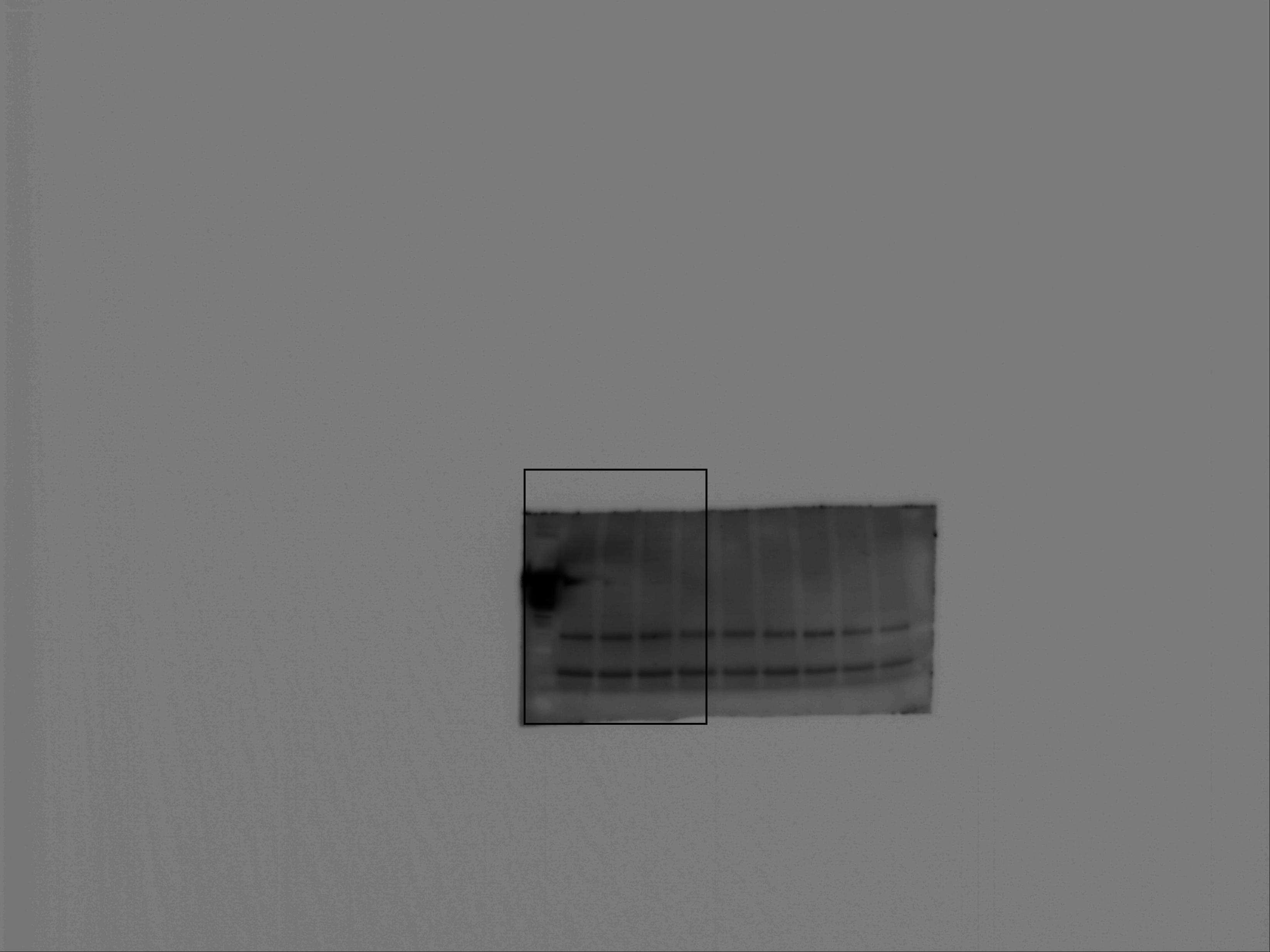

Supplement: Supplementary file 1 [file ijms-20-05816-s001.zip › ijms-618561-supplementary/p53.JPG]
